# Study on the vertical bearing performance of pile across cave and sensitivity of three parameters

**DOI:** 10.1038/s41598-021-96883-7

**Published:** 2021-08-30

**Authors:** Hui Yun Chen, Zhong Ju Feng, Tie Li, Shao Fen Bai, Cong Zhang

**Affiliations:** School of Highway, Chang’an University, Xi’an, 710064 Shaanxi China

**Keywords:** Civil engineering, Mathematics and computing

## Abstract

A new method was used to study the performance of pile across cave. This paper investigated the vertical bearing characteristics of piles cross caves using centrifugal model tests and a theoretical model of sensitivity. Twelve pile scenarios were selected, the first was a conventional pile, 24 cm long and 2.5 cm in diameter, with no karst cave as a control. In the other eleven scenarios the piles passed through karst caves of four different heights, of four different spans, and three different numbers of caves. The results reveal that increasing the height, span, and number of caves all are negative for vertical ultimate bearing capacity of piles. The axial force and unit shaft resistance of piles are great different. According to the ratios of the tip and shaft resistance, caves change the type of piles. The sensitivity of vertical ultimate bearing capacity to these factors from high to low is height, number, and span of caves. Importantly, the bearing characteristics of piles decrease faster once the height of the prototype karst cave is higher than 9 m, but decreases slowly when the cave’s span is greater than 9 m × 9 m.

## Introduction

Karst is a complex substrate, and with the stable supports required for bridge structures, the performance of piles in karst is vitally important. Three problem scenarios can occur when installing piles in terrain with karst caves: cave across pile, cave at side of pile, or cave under pile. Piles often need to pass through caves to ensure there is a stable bearing layer under pile tip for support. When the piles pass through the caves, the influence of karst cave on the bearing characteristics of piles is mainly affected by the cave’s height, span and number. A lack of rock surrounding the pile cross caves directly affects the bearing characteristics and load transfer laws of piles, which will affect the structure’s stability^1–4^.

The bearing performance of piles in karst cave has attracted much attention from scholars. Some analyzed pile’s characteristics using indoor tests, static load tests or through theoretical derivation^5–8^. Others established simulated models to study them^9,10^. Feng and He predicted the ultimate bearing capacity of piles cross the caves in vertical load with the changing height and span of the caves using the high-accuracy grey theoretical model^11,12^. Su and Xiao analyzed the adverse effects of multi-layer karst caves’ diagram on the bearing performances of piles and proposed the shaft resistance of piles gradually decrease with the increasing diagrams of caves by the indoor test and finite element analysis^13,14^. The above studied were about the bearing capacity of piles cross caves, but the load transfer mechanism of end and shaft resistance was not clear. More scholars payed attention to the calculated methods of safety roof thickness and stability of the roof when the caves were under the pile.

Dong and Jiang implemented the load-settlement laws of piles in karst area to analyze the changing of the bearing capacity of piles by static load test, and a new calculation method of safe thickness of the cave roof was obtained using theoretical deduction^15,16^. Lee and Zhang carried out the influence of five factors of underlying caves on the bearing capacity of piles using indoor test, and the results showed that with the increases of roof thickness and cave migration position, the ultimate bearing capacity increases gradually and the size order of sensitivities is cave diameter, roof thickness and equatorial radius, polar radius, cave position^17–19^. Wang established the finite limit models to study the single piles bearing force with different roof thickness, the cave’s diameter and the eccentric center of caves and carried out the roof thickness of cave was the key factor^20^. Wong and Xie studied the applicability of different types of basements in karst areas and numerically simulated four key factors of caves under piles to require the bearing characteristics, and they found that the shaft resistance was enhanced by the increasing cave’s size^21,22^. Fattah and Zhao experimentally studied the ultimate bearing force of piles under changes of two sizes of caves and established the simply supported mechanical model to get the destroy mode of the roof, when the roof is subjected to punching, shear and bending and tensile failure^23–25^. These numerous studies were all about the bearing capacity of piles cross caves or on a cave, the calculate method of the safe roof thickness and the stability of caves under the piles. However, there was very few researches about the load transfer mechanism and the shaft resistance and tip resistance of piles cross karst caves.

Centrifugal model tests were shorter test periods, lower costs, and are convenient. Furthermore, the high accuracy of centrifugal model test results has been proved by many researches^26–30^. Therefore, centrifugal model tests were used in this study to analyze the vertical bearing performance of pile across cave and sensitivity of three parameters. In this study, the effects of the cave’s height, span, and number on the bearing characteristics of piles were investigated by centrifugal tests and a theoretical model. The vertical bearing capacity were obtained under different caves. Meanwhile, the load transfer characteristics were studied under different scenarios including the axial force, unit shaft resistance and ratios of two resistances. Sensitivity of the vertical ultimate bearing pressure to above factors were calculated by a theoretical model.

## Results and discussion

**Figure 1:**
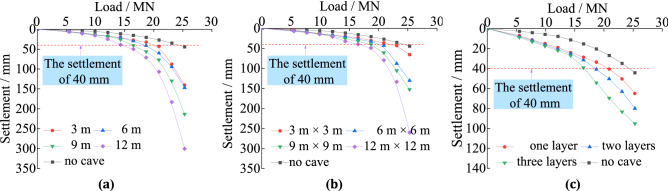
The load–settlement curves of piles under three factors: (**a**) load–settlement curves under different cave’s height; (**b**) load–settlement curve under different cave’s span; (**c**) load–settlement curve under different cave’s number;

The results were transformed into units corresponding to the prototype pile according to the similarity ratio. There are two methods requiring the ultimate vertical bearing force of piles from load-settlement curve according to the standard. The first calculates ultimate vertical bearing force as the load corresponding to the sudden change settlement. The other method is to calculate the value of 3% of the pile diameter and the value of 40 mm, then elect the smaller one to regard as the failure settlement. The load needed to produce this settlement is the bearing capacity of piles when the settlement does not mutate. Because no abrupt settlement is apparent in Fig. 1, no corresponding loads can be extracted. Besides, a settlement of 3% of the pile diameter (2 m) was 60 mm, which is greater than 40 mm, so the ultimate settlement is 40 mm and the corresponding load is the vertical ultimate bearing force.

### Analysis of bearing capacity of piles under three factors of caves

#### Load–settlement curve and bearing capacity

When the height is 3 m, span is 3 m × 3 m, and number of the karst caves is one, the vertical bearing capacities of the piles are reduced by 4.1 MN, 1.8 MN, and 4.1 MN, respectively, compared to piles with no karst cave. This shows that, in all scenarios, the ultimate vertical bearing capacity of piles passing through karst caves will be reduced.

**Figure 2:**
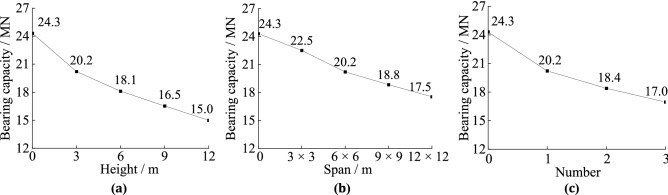
The ultimate bearing capacity of piles under three factors: (**a**) ultimate bearing capacity under different cave’s height; (**b**) ultimate bearing capacity under different cave’s span; (**c**) ultimate bearing capacity under different cave’s number.

As shown in Figs. 1a and 2a, the load-settlement curves and piles’ bearing capacity when they pass across caves of different heights can be obtained. Under identical loads, taller karst caves result in greater pile settlement. The ultimate vertical bearing force shows a gradual decreasing trend with increasing cave height. When the height of the karst caves increases from 0 to 3 m, the bearing capacity of piles reduces 4.1 MN. Because there are no material in the karst caves to provide shaft resistance to those portions of the piles. It results in the relative displacements of the piles and soil increasing. As shown in Figs. 1b and 2b, as the karst cave spans increase the settlements of the piles also increase under identical loads. The ultimate vertical bearing force decreases when the spans of the karst caves increase. However, when the karst caves’ span is greater than 9 m × 9 m, the ultimate vertical bearing capacity of piles decrease more slowly. The main reason is that there was no shaft resistance on the portions of the piles passing through the karst caves. Under a load, the instability resulting from the empty voids worsens, and this trend is more pronounced with increasing cave span. The effect of karstic span increasing on the bearing force cannot always increase. Figures 1c and 2c shows the load–settlement curves and ultimate bearing capacities of piles when the number of karst caves increases. The effect of karstic number increasing on the bearing capacity of the piles is similar to that of increasing cave height. With more layers of the karst caves, the load required to reach the same settlement depth is reduced. The ultimate vertical bearing force decreases with increased caves number. When the layer of the karst caves is 0, 1, 2, and 3, the vertical ultimate bearing capacity is 24.3 MN, 20.2 MN, 18.4 MN, 17.0 MN, respectively. The main reason is that increasing of the caves number leads to a larger amount of missing rock, so the shaft friction of the piles will decrease.

### Analysis of axial force under the three factors of caves

The shaft strain of pile foundations was obtained from the recorded data. Then the axial force on the pile foundations was calculated using Eq. (1):

1 $$ P_{A} = A_{p} E_{s} \varepsilon $$

where, *P*_*A*_ is the axial force; *A*_*p*_ is cross-sectional area; *E*_*s*_ is elasticity modulus; and *ε* is shaft strain.

#### The axial force curves under different cave height

**Figure 3:**
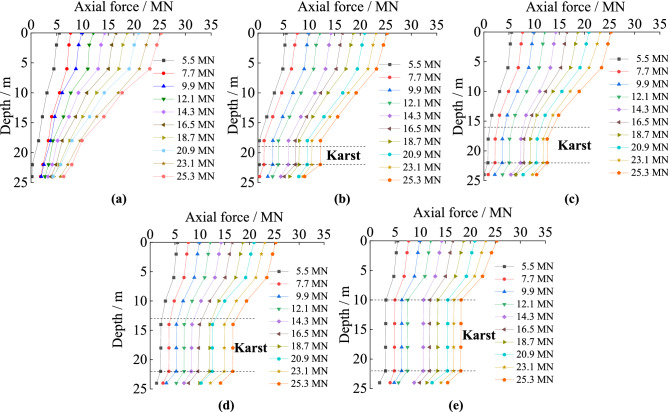
The axial force of piles with different karst cave heights: (**a**) no cave; (**b**) cave’s height is 3 m; (**c**) cave’s height is 6 m; (**d**) cave’s height is 9 m; (**e**) cave’s height is 12 m.

In Fig. 3, the pile’s axial force at the same depth gradually increases with increasing pile load, but changes abruptly at the interfaces of soil and rock. In the soil, the axial force has slowly decreasing in the pile’s length orientation, and it decreases faster when the pile was in rock. The axial force of the pile crossing no cave decreases continuously in its length orientation in rock. When there is a karst cave, the axial force of the piles changes abruptly at both the top and bottom of the karst cave. The axial force attenuation of the piles under different cave heights is similar, but the turning points are different. The axial force of the piles does not decrease in the portions passing through caves. The taller the karst cave is, the greater the axial force that is transferred to the pile bottom. The main reason is that rock provides greater shaft resistance compared to soil or empty cave. The taller the cave is, the more it is weakened by lack of lateral resistance. With larger gaps in the rock, the load transferred to rock through shaft resistance is reduced, and more load is transferred to the pile bottom, which is borne by the rock at the pile bottom.

#### The axial force curves under different cave span

**Figure 4:**
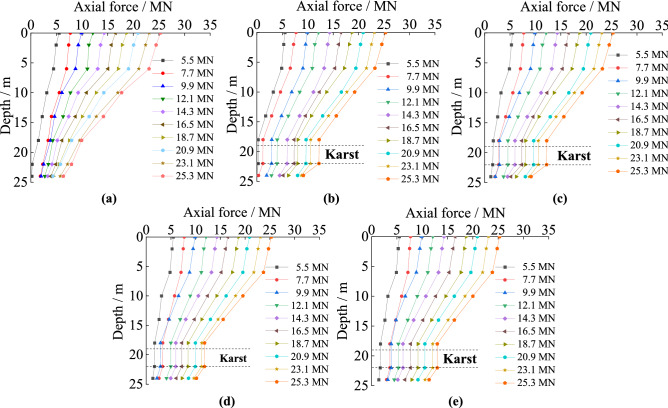
The axial force of piles with different karst cave spans: (**a**) no cave; (**b**) cave’s span is 3 m × 3 m; (**c**) cave’s span is 6 m × 6 m; (**d**) cave’s span is 9 m × 9 m; (**e**) cave’s span is 12 m × 12 m.

The trends in axial force experienced by piles in soil and rock and karst caves with varying spans are similar as shown in Fig. 4. Interestingly, under the same load, the axial forces have the same turning point despite the differences in the span of the karst caves. When the load is 9.9 MN or less, the axial forces transferred to the pile bottoms are similar, even if the spans of the karst caves are different. When the load is larger than 9.9 MN, the axial force transferred to the pile bottom increased with increasing cave span. The main reason is that the heights of the karst caves are all set to 3 m in the test, so the turning points of the axial forces all occur at the same position. In addition, increasing the span of the caves makes the empty voids collapse more easily under loads, so the load cannot be transferred to the surrounding rock through shaft resistance.

#### The axial force curves under different cave number

**Figure 5:**
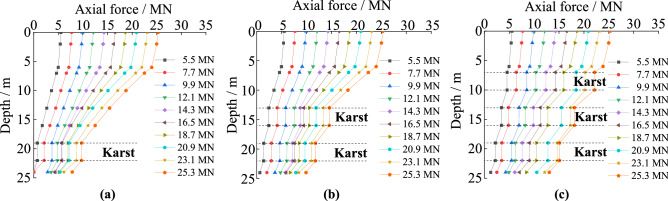
The axial force of piles with different number of karst caves: (**a**) cave’s number is one; (**b**) cave’s number is two; (**c**) cave’s number is three.

The trends of the axial force of piles in soil and rock are similar for the tests with different numbers of karst caves in Fig. 5. The difference is that under the same load, the axial forces exhibit different turning points, with caves acting as gaps where axial force does not change. The axial force law of the piles changes abruptly at the interfaces of soil and rock, and at both the top and bottom of the karst caves. With the increased number of caves, the abrupt transfers in axial force towards the pile bottom obviously increase. The main reason is that the portions of the piles in the karst caves cannot transfer load via shaft resistance into the surrounding rock. This was important because the length of the piles passing through the cave increases with increasing number of the caves, so the rock providing shaft resistance to the pile is reduced.

### Analysis of unit shaft resistance under the three factors

Equation (2) were applied to calculate the unit shaft frictions of piles after the height of pile’s section and the axial force of piles have been determined.

2 $$ P_{T} = \left( {P_{An} - P_{A(n + 1)} } \right)/\pi dh $$

where, *P*_*T*_ is unit shaft resistance of pile; *P*_*A*_ is axial force; *n* is strain gauges’ number, increasing from pile top to tip; *h* is height of pile element between adjacent components; and* d* is diameter of pile.

#### The unit shaft resistance under different cave height

**Figure 6:**
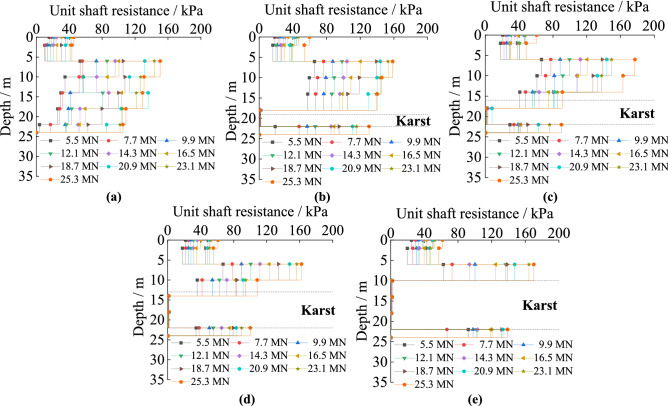
The unit shaft resistance of piles with different karst cave heights: (**a**) no cave; (**b**) cave’s height is 3 m; (**c**) cave’s height is 6 m; (**d**) cave’s height is 9 m; (**e**) cave’s height is 12 m.

Figure 6 is about the unit shaft resistance of piles passing through karst caves of different heights. It decreases in both soil and rock along the pile’s length. The unit skin friction in rock is obviously larger than that in soil. The unit shaft resistance decreases sharply when piles pass through karst caves, and it is almost zero within the karst caves. The range in which resistance goes to zero expands with increasing karst cave height. With increased cave height, the unit shaft resistance in the same soil increases. It is because rock has a larger standard value of unit skin resistance compared with soil, and the interaction between the pile and the rock requires greater forces to produce the same displacement compared to the interaction between piles and soil. Karst caves are empty voids in which no material contacts the pile, thus there is little lateral resistance provided to the pile. The reduction of materials squeezing the pile leads to increased settlement of piles, so the soil and rock around the pile provide increased resistance.

#### The unit shaft resistance under different cave span

**Figure 7:**
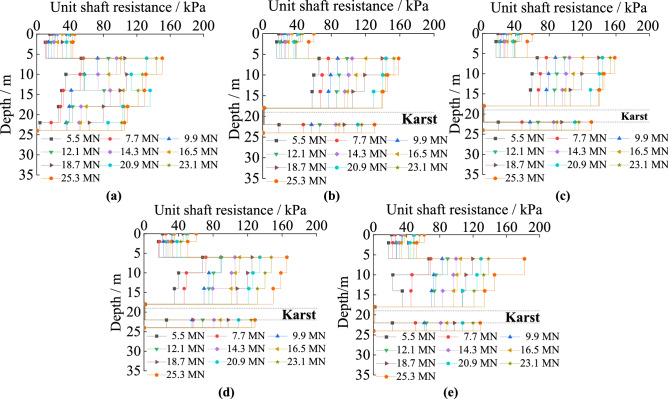
The unit shaft resistance of piles with different karst cave span: (**a**) no cave; (**b**) cave’s span is 3 m × 3 m; (**c**) cave’s span is 6 m × 6 m; (**d**) cave’s span is 9 m × 9 m; (**e**) cave’s span is 12 m × 12 m.

The unit shaft resistance of piles with five kinds of cave’s spans can be seen in Fig. 7. It decreases in both the soil and rock, and reaches its maximum at the point that the piles embed in rock. The maximum value of the unit shaft resistance is positively correlated with karst cave span. The unit skin friction decreases sharply when piles pass through the caves, decreasing to almost zero within the karst cave. Increases in cave span have little effect on unit skin friction. For cases where the karstic span is smaller than 6 m × 6 m, the unit shaft resistances are similar. When the span is greater than 6 m × 6 m, at the same depth, it increases slightly. Because the relative displacement by the pile in the soil produces downward displacement. When the karst cave is larger than 6 m × 6 m, there is a larger horizontal area where rock is missing, which leads to the karst cave easily collapsing.

#### The unit shaft resistance under different cave number

**Figure 8:**
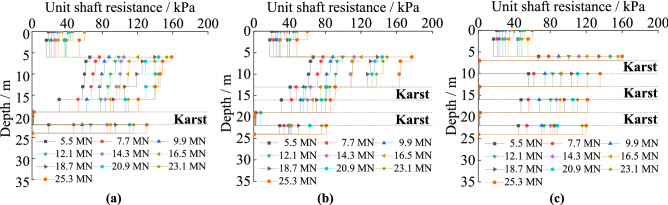
The unit shaft resistance of the piles with different numbers of karst caves: (**a**) cave’s number is one; (**b**) cave’s number is two; (**c**) cave’s number is three.

Figure 8 shows the unit shaft friction law of the piles in soil and rock when the number of karst caves increases followed that observes in piles with different cave heights. However, under identical loads, the unit shaft resistance law exhibits different turning points depending on the number of caves. The unit shaft resistance changes abruptly at the interfaces between soil and rock, and it is almost unproductive in the karst caves. The unit shaft resistance in the same soil increases as the number of caves increase. The main reason is that the shaft resistance decreases the load on the pile down its length and it increases with the increasing displacement of the pile top^31–33^. The portion of the pile within the karst cave had no contact with load-transferring material, and as such could not transfer the load into the surrounding rock via shaft resistance as the cave was empty. As shown in Fig. 1c, with the same load, the pile top settles more with increasing number of caves. This indicated that increasing the total gaps in the rock leads to increased displacement of the pile top. Hence, the unit shaft resistance increases with the increasing number of caves.

### Analysis of tip resistance and shaft resistance under ultimate bearing capacity of piles

The tip resistance of the piles under the ultimate vertical load was obtained by fitting through interpolation. The shaft resistance could be calculated by Eq. (3). The ratios could be obtained by Eqs. (4) and (5).

3 $$ Q_{si} = Q_{ui} - Q_{ti} $$

where, *Q*_s*i*_ is the shaft resistance; *Q*_*ui*_ is the ultimate bearing force; and *Q*_*ti*_ is the tip resistance; *i* is the condition number.

4 $$ \alpha_{i} = Q_{si} /Q_{ui} \times 100\% $$

5 $$ \beta_{i} = Q_{ti} /Q_{ui} \times 100\% $$

where, $\alpha_{i}$ is the ratio of shaft resistance; and $\beta_{i}$ is the ratio of tip resistance.

**Figure 9:**
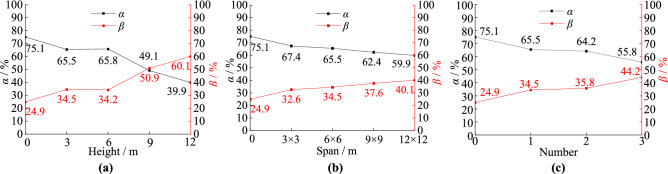
The shaft resistances’ ratios and tip resistances’ ratios: (**a**) cave’s height; (**b**) cave’s span; (**c**) cave’s number.

As shown in Fig. 9a, when the karst cave heights are 0, 3, 6, 9, and 12 m, the tip resistance’s proportions are 24.9%, 34.5%, 34.2%, 50.9%, and 60.1% and the shaft resistance’s proportions are 75.1%, 65.5%, 65.8%, 49.1%, and 39.9%, respectively. With increased karst cave height, the tip resistance of the pile shows a gradual increasing trend and the shaft resistance of the piles shows a gradual decreasing trend. With these changes, the pile type gradually changes from friction pile to end-bearing pile. The tip resistance ratio even exceeds that of the general end bearing pile. Because the section of piles that is squeezed by rock decreases as cave height increases, so less shaft resistance is exerted and more force is transferred to the pile bottom. When it surpasses 6 m, the ratio of the shaft resistance decreases rapidly. The augment of relative displacement between the piles and rock leads to the foundations receive greater shaft resistance due to the karst caves. When the cave height is 6 m, the lateral resistance peaks, but when it exceeds 6 m, the gap in the rock is too large and creates large relative displacement of the pile and rock, leading to an increase in the tip resistance.

As shown in Fig. 9b, when the karst cave span is 0, 3 m × 3 m, 6 m × 6 m, 9 m × 9 m, and 12 m × 12 m, the tip resistance’s proportions are 24.9%, 32.6%, 34.5%, 37.6%, and 40.1% and the shaft resistance’s proportions are 75.1%, 67.4%, 65.5%, 62.4%, and 59.9%, respectively. The general tendencies of the ratios are similar with the karst cave height. The ratios of tip and shaft resistance change linearly with changes in the cave’s span, with little curvature. Furthermore, the pile type gradually changes from friction pile to end-bearing pile. Because increasing the cave span does not reduce the range of the axial directional rock around the pile but does deteriorate the stability of the karst cave.

As shown in Fig. 9c, when there are 0, 1, 2, and 3 karst caves, the tip resistance’s proportions are 24.9%, 34.5%, 35.8%, and 44.2% and the shaft resistance’s proportions are 75.1%, 73.9%, 64.2%, and 55.8%, respectively. Furthermore, the pile type gradually changes from friction pile to end-bearing pile. When the cave’s number increases from 1 to 2, the ratio of shaft resistance increases slightly, but when it exceeds 2, the ratio of shaft resistance decreases rapidly. The main reason is that the lateral resistance peaks when there are 2 caves. When there were more than 2 caves, the cumulative gap in the rock is too large and creates a much larger settlement of the piles, which causes the tip resistance to become greater.

The ratios of shaft resistance of the piles passing through karst caves are obviously smaller than those that does not pass through karst caves. The main reason is that the void creates by a cave does not squeeze the piles passing through it, and as such does not exert shaft resistance on the pile.

### Analysis of parametric sensitivity analysis of bearing characteristics

The theoretical sensitivity analysis method is used to establish a model system. System characteristic (*P*) represents the vertical ultimate bearing force under the three factors, as shown in Eq. (6). The sensitivity function is shown in Eq. (7).

6 $$ P = f(x_{1} ,x_{2} , \ldots ,x_{n} ) = \varphi_{i} (x_{i} ) $$

7 $$ S_{i} (x_{i} ) = \left| {d\varphi (x_{i} )/dx_{i} } \right| \cdot x_{i} /P\;\;i = {1},{ 2}, \, \ldots n $$

where, *P* is system characteristic; *x*_*i*_ is height, span, or number of karst caves; and $S_{i} (x_{i} )$ is sensitivity.

The datum parameter set is the overlapping parameter of the karst cave in the test. According to the engineering manual and the most common karst cave parameter suggestions in the engineering literature. Table 1 is about the datum parameter set.

**Table 1:** The datum parameter set.

Height/cm	Length/cm	Number
3	6	1

The system characteristic curves *P–x*_*i*_ are shown in Fig. 7. The formulas for *P* and *H*, *P* and *L*, and *P* and *N* are established by Origin fitting, as shown in Table 2.

**Table 2:** The functions of the system characteristics.

*x*_i_	Functions	Accuracy of fitting *R*_2_
Height	$\varphi_{1} (x_{1} ) = - 0.006x_{1}{^{3}} + 0.1527x_{1}{^{2}} - 1.7454x_{1} + 24.297$	0.9997
Length	$\varphi_{2} (x_{2} ) = 0.0018x_{2}{^{3}} - 0.0169x_{2}{^{2}} - 0.6183x_{2} + 24.343$	0.9975
Number	$\varphi_{3} (x_{3} ) = - 0.3162x_{3}{^{3}} + 2.086x_{3}{^{2}} - 5.8647x_{3} + 24.311$	0.9999

**Figure 10:**
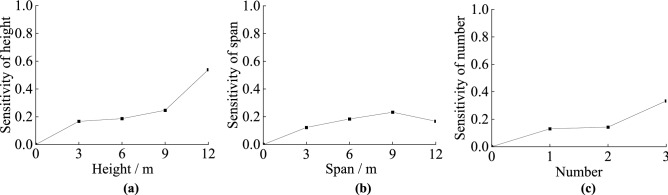
The sensitivity curves: (**a**) height of karst caves; (**b**) span of karst caves; and (**c**) number of karst caves.

According to the Eq. (7), the sensitivity functions and sensitivity factors of the three factors could be obtained, as shown in Table. 3. The sensitivity is the calculated using the functions and the sensitivity curves are shown in Fig. 10.

**Table 3:** Sensitivity function and sensitivity factors for the three factors.

*x*_i_	Sensitivity functions	Sensitivity factors
Height	$S_{H} (x_{1} ) = \left| {\frac{{ - 0.1527x_{1}{^{2}} + 3.4908x_{1} - 72.891}}{{ - 0.006x_{1}{^{3}} + 0.1527x_{1}{^{2}} - 1.7454x_{1} + 24.297}} + 3} \right|$	0.1676
Length	$S_{L} (x_{2} ) = \left| {\frac{{0.00169x_{2}{^{2}} + 1.2366x_{2} - 73.039}}{{0.0018x_{2}{^{3}} - 0.0169x_{2}{^{2}} - 0.6183x_{2} + 24.343}} + 3} \right|$	0.1229
Number	$S_{n} (x_{3} ) = \left| {\frac{{ - 2.086x_{3}{^{2}} + 11.7294 - 72.933}}{{ - 0.3162x_{3}{^{3}} + 2.086x_{3}{^{2}} - 5.8647x_{3} + 24.311}} + 3} \right|$	0.1307

The sensitivity of the ultimate vertical bearing force of the piles increases with increasing cave height. However, when the cave’s height is less than 9 cm, the ultimate force is not sensitive to the cave height. Only when it exceeds 9 cm dose the sensitivity increases significantly. The main reason is that when karst caves are higher than 9 cm, the continuous gap in the rock around the pile becomes too large, and the pile is more prone to fracture in the cavity.

The sensitivity of the ultimate vertical bearing force with increasing karst cave span initially increases and then decreases. The sensitivity increases linearly with increasing span when it is less than 9 cm × 9 cm, but it decreases significantly when the span is greater than 9 cm × 9 cm. It is because that the cave becomes more unstable due to the increasing cave span. However, even if the karstic span augments infinitely, but its height is constant, the rock that squeezes the pile does not decrease. Therefore, when the karst cave’s span exceeds a certain value, its increase no longer has a large effect on the ultimate capacity of piles.

The sensitivity trend is similar to that observes with increasing cave height. However, the difference is that the sensitivity of ultimate vertical bearing capacity to cave height is greater than to cave number. When the number of caves exceeds 2, the sensitivity increases significantly. This is because the layers of rock between each layer of cave have a certain thickness, this means there are no large continuous gaps in the rock around the pile. When the number of caves exceeded 2, there are too many transitions between rock and empty void, and too much missing rock around the pile.

The size order of the sensitivity of ultimate vertical bearing capacity to above parameters is height, number, span.

It is worth noting that the theoretical sensitivity analysis method is a systematic method that utilizes mathematical statistics. The degrees of the effects of the height, span and number of karst caves on pile’s bearing capacity can be quickly obtained using this method. Through this analysis the critical cave parameters affecting the pile can be identified to provide reference for the design of piles in karst areas. However, the datum parameter set and the range of the three parameters of caves are often based on geological exploration data and engineering experience, so it is possible that the accuracy of the sensitivity analysis result is influenced by the number of data samples, so the samples should be as comprehensive as possible.

## Conclusions

In this study, the effects of the cave’s height, span, and number on the bearing characteristics of piles were investigated by centrifugal tests and a theoretical model. The conclusions of this study are as follows:

1. Increased karst cave height, span, and number all decrease the ultimate bearing capacity of the piles. It decreases rapidly when prototype cave height is greater than 9 m, and decreases slowly when the span of the cave is greater than 9 m × 9 m. When the size of the cave exceeds the above range, the damage of piles must be considered cautiously. The sensitivity of the vertical ultimate bearing capacity to above three factors from high to low is height > number > span. The influence of cave height on pile should be paid more attention in engineering.
2. The axial force of the pile decreases very slowly over portions of the piles within the karst caves and rapidly in portions of pile surrounded by rock. The main reason is that no shaft resistance is exerted on the pile in the cave cavities.
3. The unit skin friction decreases sharply when the piles pass through karst caves, and it is almost zero within the karst caves. With increased cave height, the unit shaft resistance of the piles in same soil increases. The cave span’s increasing has little effect on the unit shaft resistance that increases as the number of caves increases in the same depth.
4. The shaft resistance’s ratio under the ultimate bearing force of the piles passing through the karst caves are obviously smaller than the piles do not pass through karst caves. With increased karst cave height, span, and number, the ratio of tip resistance gradually increases, meanwhile the shaft resistance decreases gradually. The pile type gradually changes to end-bearing pile. The shaft resistance will not keep increasing with the size and number of the karst caves increasing. Large loss of lateral resistance caused by caves should be considered in engineering, which will affect the bearing capacity of piles.

## Materials and methods

### Experimental equipment

**Figure 11:**
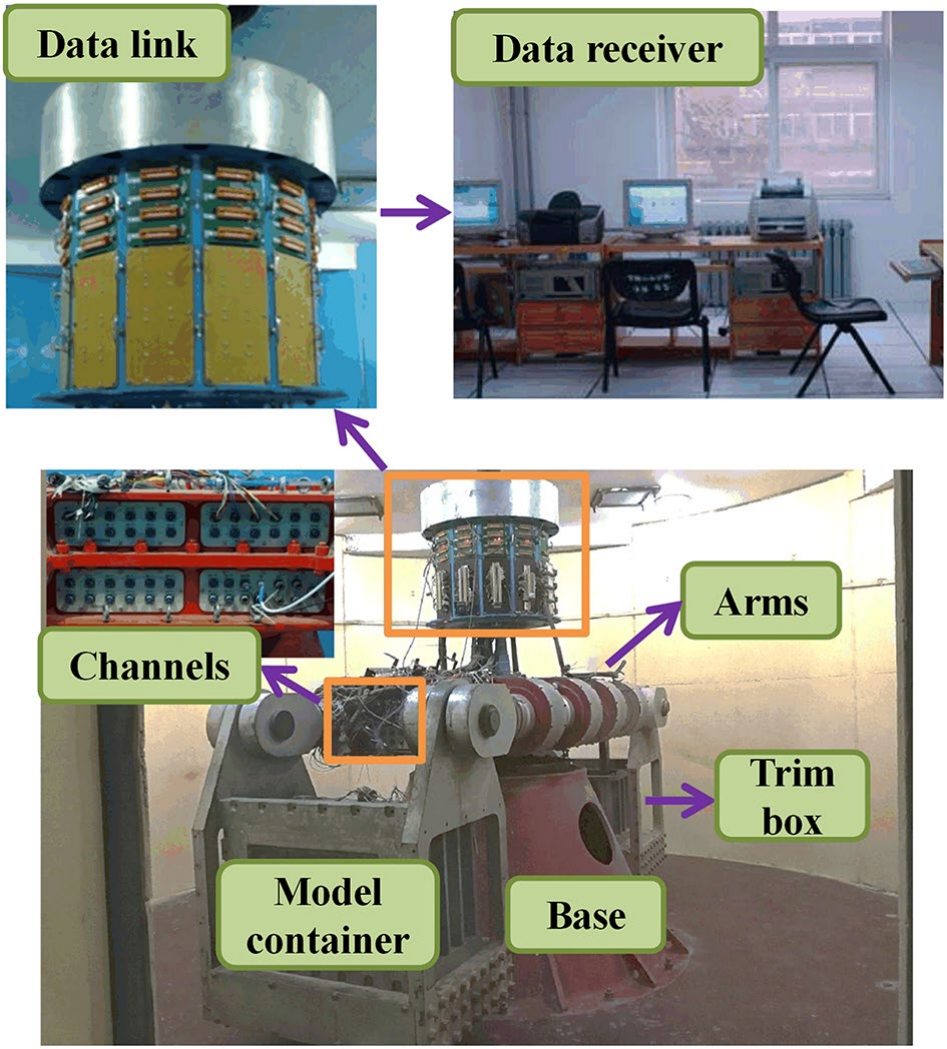
TLJ-3 type centrifuge.

A TLJ-3 geotechnical centrifuge with an acceleration range of 10–200 g was used in this experiment, shown in Fig. 11. The specification is 60 g⋅t, which means it could take 1000 kg (total weight of the model container and the model) at an acceleration of up to 60 g. The centrifuge collects data once per second and displays it directly on the data workstation through 40 channels. The centrifuge itself uses the beam type construction with a stable base supported in the middle. The model container with the model and the balancing box are placed symmetrically on either side of the beam. The length, width and height of steel model container is 70 cm, 36 cm, or 50 cm, respectively. Table 4 shows the similarity ratios of the main parameters of the test.

**Table 4:** Similitude ratios (the ratio is n).

Parameter	Ratio
Height/length	1∶n
Settlement	1∶n
Stress	1∶1
Strain	1∶1
Force	1∶n^2^
Quality	1∶n^3^

### Samples preparation

#### Piles and caves

The similarity ratio was set to be 100 considering the size of centrifugal box and prototypical pile. At present, commonly used materials for model piles are steel pipe, aluminum pipe, PVC pipe. These materials solve the problems of long production time and unstandardized strength of reinforced concrete cast-in-place model piles. The prototypical pile was simulated by aluminum pipe in this experiment, and the pipe has a Young’s modulus of 46 GPa. According to the similarity principle, the prototype’s compressive stiffness controls the model’s diameter as shown in Table 5.

**Table 5:** Characteristics of model and prototype.

Pile	Parameter				
	Length(mm)	Outside diameter(mm)	inner diameter(mm)	Young’s modulus (GPa)	Similarity ratio
Model	240	25	19	47	1
Prototype	24,000	200	–	30	100

**Figure 12:**
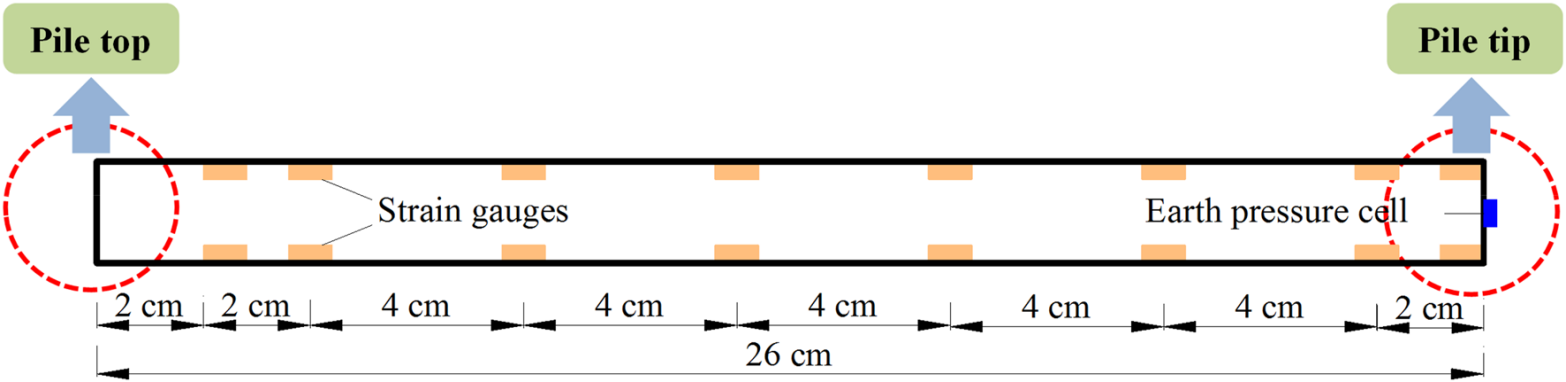
Layout diagram of test components.

**Figure. 13:**
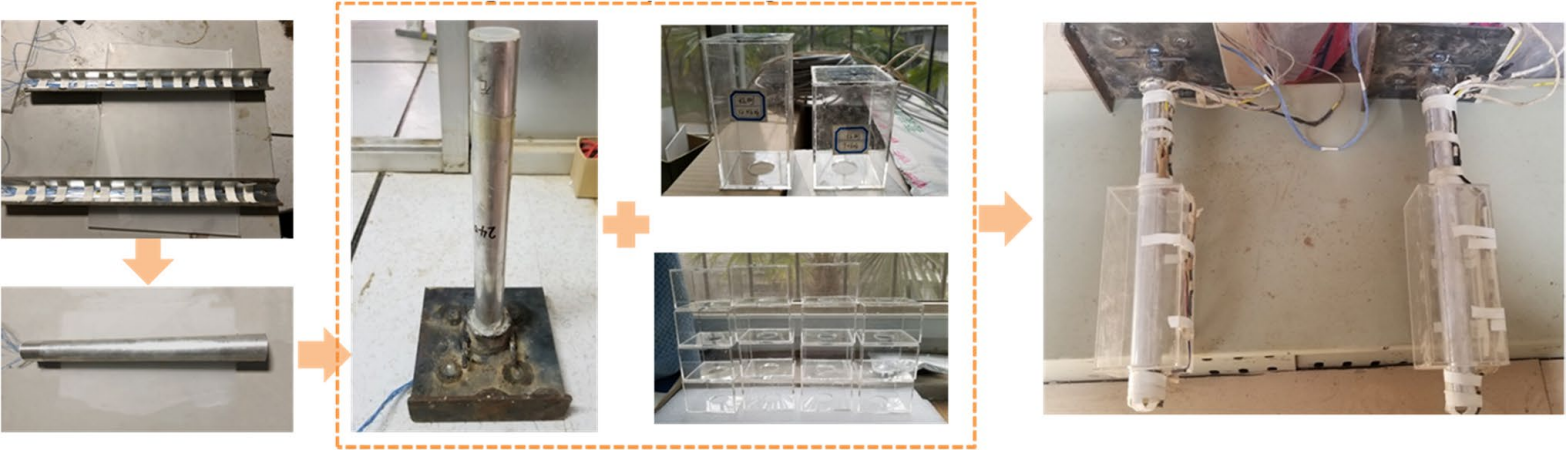
Model piles and model karst caves.

The strain gauges were fixed to the model piles cut along the axis of pipe to make sure the reliability as shown in Fig. 12. The upper 2 cm of the model piles was reserved for connecting with the loading platform. The lengths of the model piles were 26 cm, but the actual length in the substrate was 24 cm, so the distance of the top group of the components to pile top were 2 cm. The other groups were uniformly distributed along the vertical direction of model pile. After installing strain gauges, the model piles were repaired with epoxy resin. The surfaces of the piles were sanded with sandpaper to return the bearing performance as close to those of the prototype as possible. Coins were used to seal the bottom of the model piles. Organic glass boxes with 2 mm thick walls were used to approximate empty caves because of their low strength. The model piles passed through the reserved holes in the upper and lower roof of the caves and were fixed in the designed position of the model caves (Fig. 13).

#### Rock and soil

**Figure 14:**
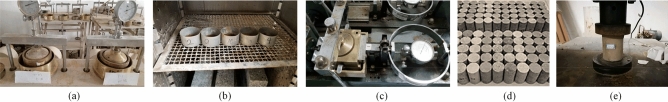
Physical property tests: (**a**) oedometer test; (**b**) moisture content test; (**c**) direct shear test; (**d**) specimens with different proportions; (**e**) compression tests.

It is extremely difficult to use undisturbed soil in the centrifugal model tests, so loess and artificially prepared rock were used to simulate the overburden and weathered rock in this test. As Fig. 14a–c, the oedometer test could test out the compression modulus. The moisture content test could get the moisture content. The direct shear test characterized the shear parameters. Cement and gypsum acted as bonding agents between the materials, they were widely used in the simulation of failure and deformation of rock. Soil was used to control the unit weight of the mixed material. The initial ratio of the cement, gypsum, water, and soil was obtained from previous studies and experience^34–37^. The samples were built and cured. The strength and deformation characteristics of the samples were obtained by repeated compression tests (Fig. 14d,e). According to the similarity ratios (Table 4), the Young’s modulus and the cohesive forces of the porotype rock, the proportions of the four materials were adjusted gradually until the strength and deformation characteristics fit the desired performance for simulating weathered rock. Then the optimal ratio of cement, gypsum, water, and soil was 1:0.5:1.1:0.8. Physical properties of soil and simulated rock can be seen in Table 6.

**Table 6:** Physical properties of soil and simulated rock.

Name	Density *ρ* (g/cm^3^)	Young’s modulus *E*_s_ (MPa)	Water content *ω* (%)	Cohesive force *c* (kPa)	Angle of internal friction *φ* (°)
Soil	1.8	6.7	20.5	15.9	15.1
Simulated rock	2.3	23.5	–	27	28

### Test process

**Figure 15:**
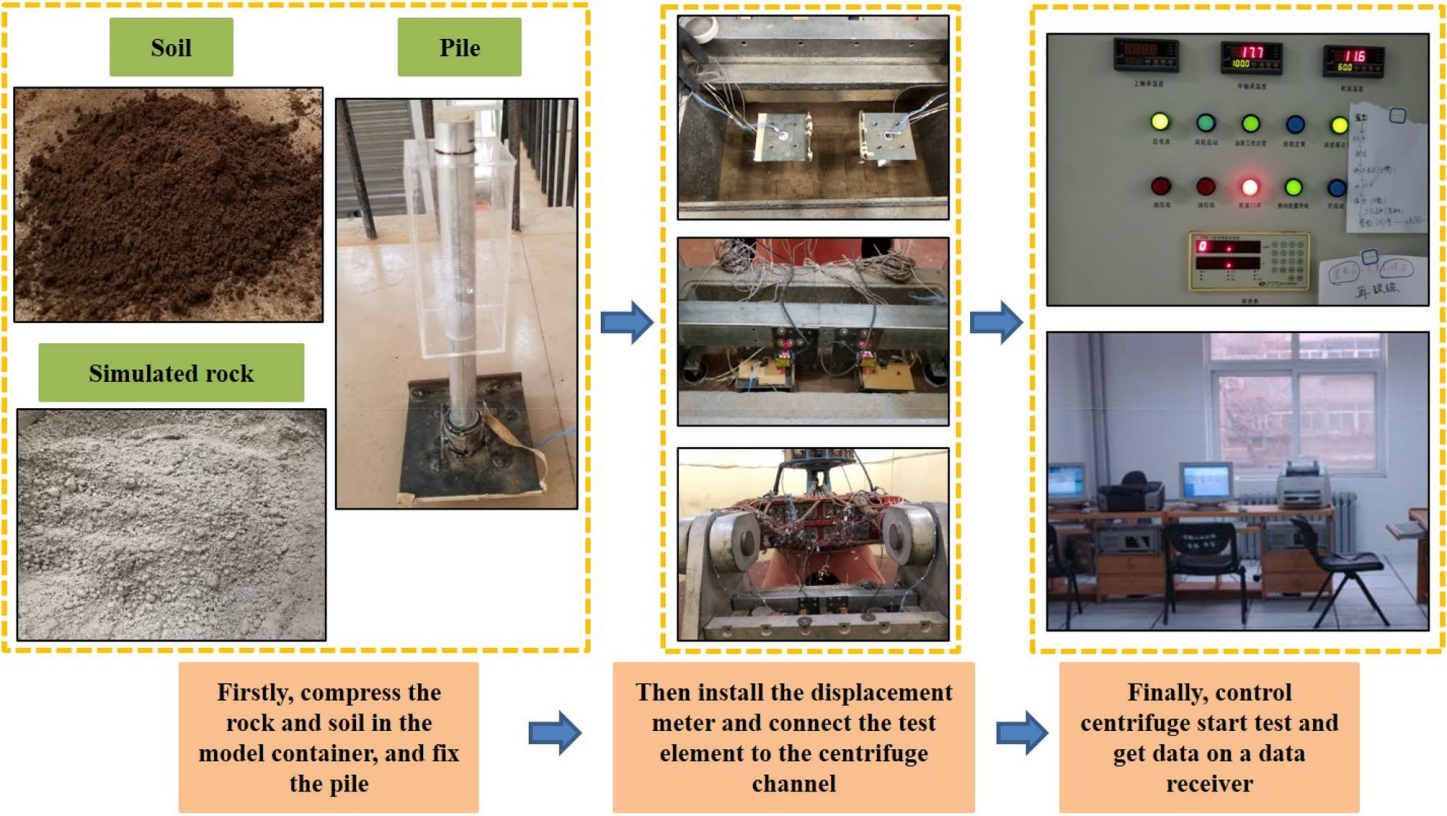
Test process.

The centrifuge model test processes can be seen as follows (Fig. 15).

**Figure 16:**
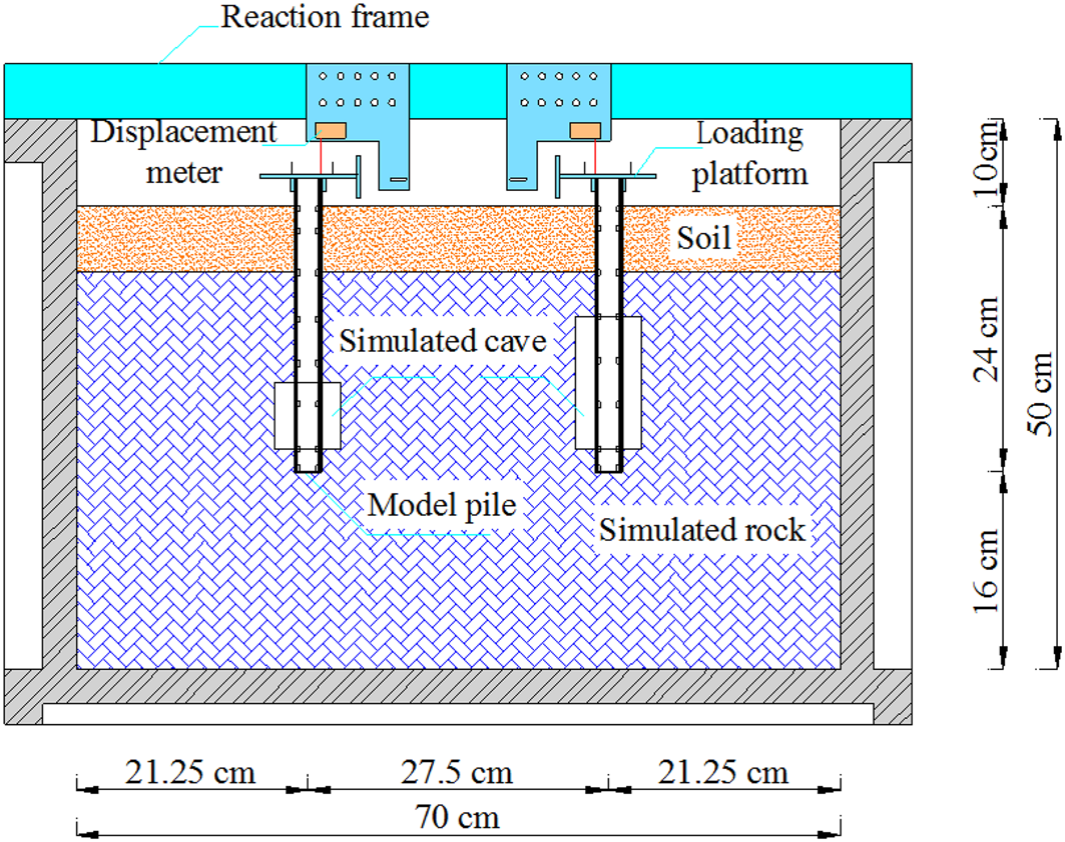
Model schematic.

1. According to Table 6 each layer of simulated rock and soil was compressed into 2 cm to ensure adequate compaction. According to the size of the model container, it was calculated that 11,592 g of material was required for each layer of simulated rock and 9072 g of material was required for each layer of soil. Firstly, 11,592 g prepared simulated rock was weighed out and evenly spread in the model container, then it was compressed to 2 cm using a vibrator. This process was repeated 8 times. Then the model piles were fixed in predetermined positions, and the above steps were repeated 9 additional times. The positions of the model piles are shown in Fig. 16. In the width direction, the pile was located in the middle. Finally, three separate 9072 g soil layers were weighed, spread, and compressed on the top of the experimental substrate.
2. The model container was weighed, hoisted onto the centrifuge, and fixed in place with bolts. Finally, the trim box was filled to the same weight as the model container.
3. The reaction frame was connected to the model container. The displacement meter was fixed to the reaction frame and the settlement of the model top could be measured. The strain gauges and earth pressure cell were connected to the data channels.
4. There were ten levels of load. The first level was the platform’s weight, and each level from the second to the tenth was increased by 220 N. In the test, two pieces of iron were added to each level and placed symmetrically on the platform.
5. The centrifugal test was conducted at a constant speed at 100 g of acceleration (100 g) for 5 min. The load was increased after one loading period ended, then the centrifuge was closed and cycled again at 100 g for 5 min. Data were collected once per second and transmitted to the data receiver.

### Experimental conditions

The parameters that are manipulated included the height, the span, and the number of karst caves that interact with piles. There are twelve total experimental treatments, including one with piles without karst caves (control), four different heights (3, 6, 9, 12 cm), four different spans (3 × 3, 6 × 6, 9 × 9, 12 × 12 cm × cm), and three different numbers of caves (1, 2, or 3 caves). When the karstic number is manipulated, the size of each karst cave layer is 3 cm × 6 cm × 6 cm (the height is 3 cm). The heights of the caves are measured in the axial direction of the pile. The spans of the caves are measured in two directions perpendicular to the axial direction of the pile.

## Data Availability

The data used to support the findings of this study are included within the article.
